# Administration, Billing, and Payment for Pharmacy Student-Based Immunizations to Medicare Beneficiaries at Mobile Medicare Clinics

**DOI:** 10.3390/pharmacy7010022

**Published:** 2019-02-25

**Authors:** Joseph A. Woelfel, Edward L. Rogan, Rajul A. Patel, Winnie Ho, Hong Van Nguyen, Emily Highsmith, Claire Chang, Nhat-Thanh Nguyen, Morgan Sato, Daniel Nguyen

**Affiliations:** Department of Pharmacy Practice, Thomas J. Long School of Pharmacy, University of the Pacific, Stockton, CA 95211, USA; jwoelfel@pacific.edu (J.A.W.); rpatel@pacific.edu (R.A.P.); w_ho1@u.pacific.edu (W.H.); h_nguyen40@u.pacific.edu (H.V.N.); e_highsmith@u.pacific.edu (E.H.); c_chang15@u.pacific.edu (C.C.); n_nguyen68@u.pacific.edu (N.-T.N.); m_sato4@u.pacific.edu (M.S.); d_nguyen36@u.pacific.edu (D.N.)

**Keywords:** immunization programs, mobile health units, experiential learning, billing, healthy people 2020

## Abstract

Training student pharmacists to administer vaccinations requires a substantial investment in vaccines, supplies, and time. Few schools of pharmacy seek out or receive any reimbursement for the provision of vaccines, despite the fact it is a covered service. This study sought to implement, deliver, and demonstrate an innovative, financially sustainable curriculum-based immunization program by trained pharmacy students as part of their experiential learning. Thirty-nine community health clinics targeting Medicare beneficiaries were conducted throughout Northern/Central California during Medicare’s fall open enrollment periods between 2014–2016. American Pharmacists Association (APhA)-trained student pharmacists (under licensed pharmacist supervision) administered 1777 vaccinations. Vaccines were billed via a secure Health Insurance Portability and Accountability Act of 1996 (HIPAA)-compliant web-based portal. The total net income was $11,905 and $8032 for 2015 and 2016, respectively. Return on investment was greatest for the influenza vaccine > Tdap > pneumococcal. Pharmacy students are already being trained to provide immunizations and can utilize their skills to deliver financially viable public health programs.

## 1. Introduction

Vaccinations are a well-known, cost-effective, way to reduce morbidity and mortality. For each group of individuals born in the same year (birth cohort) that is properly vaccinated, the following could be prevented; 14 million cases of disease, 33,000 deaths, and almost $10 billion in direct health care costs. However, approximately 42,000 adults in the United States still die from vaccine-preventable diseases each year [1]. 

The Department of Health and Human Services initiated the Healthy People 2020 program with the intent to promote the health of Americans by encouraging collaboration across communities, empowering individuals, and measuring the impact of these interventions [1]. One notable goal is the improvement of vaccination rates. Healthy People 2020 seeks a goal of at least 90% of adults 65 years of age and older to be vaccinated against both influenza and pneumococcal disease [1]. In 2014, data released by the Centers for Disease Control and Prevention (CDC) showed that approximately 70% of seniors 65 years old and over were vaccinated against influenza and approximately 61% against pneumococcal [2].

Inadequate vaccination rates could be a result of multiple factors, including a lack of perceived value, insufficient information and education, fear or opposition to vaccines, cost, accessibility, and operational or systemic barriers [3]. Despite the fact that Medicare is the primary insurance for most seniors, 36% to 71% of general internists and family physicians reported a lack of knowledge of Medicare vaccine coverage [4].

Lower adult vaccination rates may also be due to the lack of immunization programs that support and promote adult vaccinations. Vaccines for Children (VFC), a federally funded program through the CDC, provides discounted vaccines to agencies that then distribute them to doctors’ offices and public health clinics. The VFC program has proven successful in improving childhood vaccination rates. Further contributing to the disparity in vaccination rates between children and seniors is the fact that many older patients may be unaware of the importance of indicated vaccines, a pattern first observed during adulthood [3]. The lack of proper education on immunizations remains an issue. Studies that examined attitudes toward the influenza vaccine found that the main concern among those eschewing the vaccine was the belief they would get influenza from the vaccination or experience adverse effects [5]. Barriers to vaccination uptake among patients 65 years and older include a lack of awareness of the need to get vaccinated and the perceived belief that they were unlikely to catch “the flu” [5]. Interestingly, greater than half of unvaccinated patients studied did not know that the entire cost of the influenza vaccine was a covered Medicare benefit [5]. Increased understanding of this benefit should encourage greater uptake of the influenza vaccine by older patients. The study also found that patients who were recommended immunizations by a healthcare professional were more likely to be vaccinated. Immunization rates can be increased due to pharmacists’ easy accessibility and their ability to dispel vaccine safety concerns and explain the risks of being unvaccinated. [5].

Expanding the provision of vaccines into non-traditional settings, such as worksites and pharmacies, has been proven to be a cost-effective way to improve adult vaccination rates. Prosser reported that the mean cost of providing vaccines was 40%–60% lower in non-traditional settings when compared to traditional settings, such as physician offices [6]. Moreover, it was found that the cost of preventing an episode of influenza was $90 in a pharmacy setting (e.g., when a pharmacist provides the vaccine), $210 in a mass vaccination setting, and $870 in traditional settings. Providing vaccines in non-traditional settings helps increase patient convenience, improves vaccination coverage rates, and is cost-effective [6].

Since pharmacists can play a major part in increasing community vaccination rates, student pharmacist training is crucial to preparing tomorrow’s pharmacists to take up this vital public health role. As of June 2017, 67/135 schools of pharmacy throughout the United States are certified to provide the American Pharmacists Association’s (APhA’s) Pharmacy-Based Immunization Delivery program to their students [7]. The APhA’s Pharmacy-Based Immunization Delivery Program is a national training certificate program employed to educate pharmacists on how to become vaccination providers [7]. After completion of this training, and subject to their state’s scope of practice laws, student pharmacists are able to provide vaccinations to patients. Previous research by Turner and colleagues has shown that the provision of vaccines by student pharmacists during introductory pharmacy practice experiences can enhance students’ vaccine knowledge and increase students’ self-confidence in the administration of vaccines [8]. Chou and colleagues showed that student pharmacist consultation can improve patient perceptions and attitudes towards receiving vaccinations, further increasing adult vaccination uptake and changing the public’s view of the pharmacist’s role in preventive health [9]. The provision of vaccines by student pharmacists also helps schools of pharmacy satisfy accreditation standards for disease prevention promotion set by the Center for the Advancement of Pharmacy Education [10].

While the majority of pharmacy schools across the county have pharmacy students provide vaccinations to the community, few provide vaccines other than influenza, and even fewer bill for provided vaccines. A previous study at The University of Oklahoma College of Pharmacy described the integration of introductory and advanced pharmacy practice experiences within campus-based influenza clinics to provide vaccinations to faculty, staff, and their families [11]. They reported that billing through their on-campus retail pharmacy generated a net income for their clinics [11]. 

Even though students from schools of pharmacy have been providing vaccinations for some time now, little literature exists on the provision of any other vaccines besides influenza being provided by student pharmacists. This may be due to the fact that the influenza vaccine is considerably less expensive to provide than all other available vaccines, and thus more financially feasible given limited school budgets. 

This study details the creation and application of a curriculum-based, financially self-sustaining pharmacy school program designed to increase vaccination rates in ambulatory Medicare beneficiaries while providing an experiential learning environment. 

## 2. Materials and Methods

During the fall Medicare open enrollment periods from 2014–2016, the University of the Pacific School of Pharmacy & Health Sciences held thirty-nine Mobile Medicare Clinics in 10 different cities throughout Northern/Central California. Beneficiaries at each clinic site were able to take advantage of multiple health services/screenings, including: Medicare Part D plan reviews, Medication Therapy Management (MTM), health screenings, and immunizations.

The following vaccines were available at each clinic location: influenza (TIV and/or QIV); Pneumovax 23 (PPSV 23); Prevnar 13 (PCV13); and the tetanus, diphtheria, and pertussis vaccine (Tdap). The influenza vaccine was both purchased by the School and also donated by multiple different county public health departments. All other vaccines and supplies were purchased by the School. During each MTM session, assisted beneficiaries were asked a series of questions so as to ascertain their vaccination history. State vaccination registries and immunization providers were contacted when patients were unsure of their vaccination history. To improve convenience, patients were able to receive vaccinations at one of two locations at each clinic site: (1) a designated vaccination station or (2) the table at which they were receiving the MTM intervention. Walk in patients not receiving MTM services could also receive vaccinations. 

Beneficiaries expressing interest in receiving any of the available vaccines provided personal information (including their Medicare ID number) and were asked a series of standard APhA vaccine screening questions, such as those relating to allergies, previous reactions to vaccines, pregnant or could be pregnant, etc. All answers were reviewed by a licensed, immunization-certified pharmacist and vaccine appropriateness was determined. School faculty and/or staff would verify beneficiary eligibility and financial responsibility for Part B (influenza or pneumococcal vaccine) or Part D (Tdap) vaccines using TransactRx, an internet-based billing and integrated claims processing platform. TransactRx provides real-time patient eligibility and on-line billing and payment for all covered Medicare Part B and D vaccines.

If the beneficiary was eligible for Part B billing of the influenza or pneumococcal vaccines, there was no co-pay sharing. However, if the beneficiary was eligible for Part D billing of the Tdap vaccine, the co-pay amount would be communicated to the patient and collected if they were interested in receiving the vaccine. Co-pays were collected via cash, personal check, or credit card using a University-approved PCI-compliant electronic payment system (CashNet). Vaccine doses that could be successfully billed were drawn from the School’s purchased supply. The Public Health influenza vaccine was used whenever an uninsured or unqualified beneficiary needed vaccination. ‘Vouchered vaccines’ were non-billable (e.g., Public Health donated vaccine, vaccines subsidized through grant funds, or vaccines given away to the community) administered vaccines. Once vaccine billing was initiated and an individual dose prepared, a certified student pharmacist administered the vaccine under the supervision of a pharmacist preceptor and provided the beneficiary with necessary paperwork (Vaccine Information Sheet (VIS) and proof of vaccination).

The number and type of vaccines provided at each mobile clinic were recorded. Return on investment (ROI) was calculated as follows: ROI = [(Total revenue − Total Cost)/(Total Cost)] × 100. Along with ROI, net income was computed each season from TransactRx billing software reports. Descriptive statistics (e.g., absolute frequency for types of vaccinations administered/billed and average ROI for each vaccination type provided) were calculated and reported for these items.

Students received introductory pharmacy practice experiences (IPPE) credit as part of a required health care outreach class that contributed approximately 8% of their total required hours. Vaccinations were coordinated by a student group, Operation Immunization, and were responsible for bringing supplies, set up, and other logistics necessary for a mobile health clinic. Additional students enrolled in the outreach class signed up to participate in the vaccination or one of the other health screenings being provided at the clinic. Students were not required to spend a specific amount of time per each station, but were required to stay at the same station for which they signed up for the duration of that event. Each event, on average, had two students from Operation Immunization and four to eight students administering vaccines. Students participated in multiple health care outreach events to acquire the required number of hours. 

This study was approved by the University of the Pacific’s Institutional Review Board.

## 3. Results

**Figure 1:**
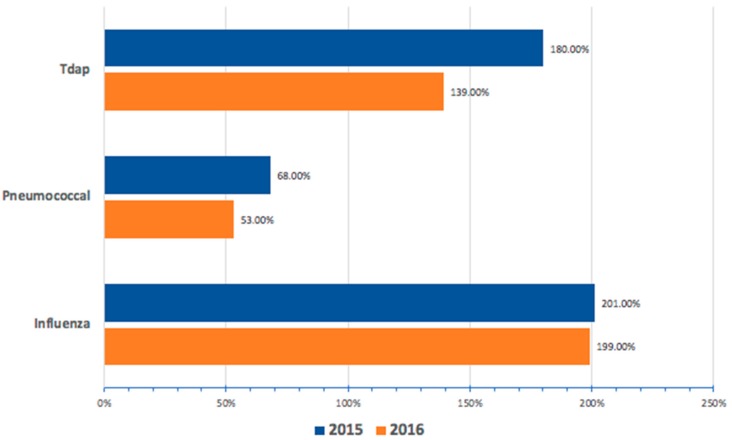
Return on Investment for Each Vaccine from 2015–2016.

A total of 4083 beneficiaries were served at our Mobile Medicare Clinics from 2014–2016. Senior immunizations were a major part of the services provided. Overall, 1777 vaccines were administered during the study time period. Table 1 identifies the type and number of vaccines administered and billed at clinic sites from 2014–2016. As shown in Table 1, about two out of three of the vaccines provided during the study period were for influenza. Of the 1777 administered vaccines, 1345 (75.7%) were billed through TransactRx (Table 1); 832 (68.2%) influenza, 449 (99.6%) pneumococcal, and 64 (60.4%) Tdap. As shown in Table 1, 629 vaccines in 2014 were billed through an independent partnering pharmacy as a proof of concept. In 2015 and 2016, 411 and 305 vaccines, respectively, were billed directly using the TransactRx platform. A total of 432 vouchered vaccines were provided during the study period; 388 (89.8%) of which were for influenza. Figure 1 highlights the ROI for each provided vaccine in 2015 and 2016. The ROI was greatest for the influenza vaccine > Tdap > pneumococcal. The total net income was $11,905 and $8032 for 2015 and 2016, respectively (data not shown). 

## 4. Discussion

The present study describes a new, fiscally self-sustainable vaccination program in which trained student pharmacists provided vaccinations to Medicare beneficiaries in community settings as part of their IPPE. Our study describes a program that utilizes a live-claim processing platform (e.g., TransactRx) for vaccines at community-based health clinics that can: (1) provide students with practical experience of immunization administration, coverage, and billing; (2) help increase vaccination rates of older adults and other Medicare beneficiaries; and (3) generate income for the provision of such services by a school of pharmacy. The above criteria set up a true “win-win-win” proposition for the target population, pharmacy students, and the school of pharmacy. 

During the fall of 2015 and 2016, successful Medicare vaccine billing generated a total net income of $19,937 for our School of Pharmacy. The generation of this net income was the first step in building an effective community outreach program in which students delivered essential and needed services in a budget-neutral way for our School. Profits from the program are used to purchase vaccines and supplies for the next season. The vaccine billing model discussed here can be duplicated at other schools of pharmacy throughout the country, with the ability to increase the types and numbers of vaccines that students can provide to their communities. 

While the majority of pharmacy schools across the county have pharmacy students providing vaccinations to the community, few provide vaccines other than influenza, and even fewer bill for provided vaccines. Our study adds to the current literature in that it describes the structure, implementation, billing, and benefit of vaccination services in a mobile, community-based setting. Utilization of a web-based claims processing platform (e.g., TransactRx) allows vaccine administration and billing in areas outside of a traditional school/university’s campus or clinic. Using an on-line claims billing service also allows patient eligibility to be checked and billed in real-time, virtually eliminating billing denials and potential loss of revenue. Furthermore, implementation of such a system can be used by any school of pharmacy across the country, regardless of whether they have an on-site medical center or pharmacy. 

Program models such as ours present schools of pharmacy a means to help fulfill the various requirements of accrediting bodies and national objectives for improving public health. In 2011, the American Association of Colleges of Pharmacy, encouraged by the Healthy People 2020 curriculum task force, instituted a new requirement in which core public health concepts were to be integrated into every pharmacy school curricula (objective ECBP-17) [12]. Additionally, our program model also helps to satisfy the Healthy People 2020’s Immunization and Infectious Diseases objective of increasing the vaccination rates of adults 65 years of age and older (Objectives IID-12.7 and IID-13.1) [13]. 

The return on investment for the three vaccines we provided varied according to the type of vaccine administered. The influenza vaccine was the highest, with about a 200% ROI. Of the three, the influenza vaccine was the most inexpensive and had the highest margin. The influenza vaccine is indicated to be administered annually and can be a continuous source of revenue for a school-based immunization clinic. The other vaccines, pneumococcal and Tdap, had a lower ROI and are only administered as a one-time dose or as a booster. The number of these vaccines declined notably after the initial year because many of our clinics are held at the same locations and draw many of the same patients every year. These vaccines did not function as a major source of revenue, but their availability was essential to providing comprehensive vaccination services to an ambulatory Medicare population with a vaccination rate below the goals of Healthy People 2020.

Some limitations of our study were that students at the time were not specifically trained to counter vaccine hesitancy from patients and that cost/coverage was a main factor impacting patients’ acceptance of vaccination. Preparing students to better address vaccine myths and misinformation could further improve vaccination rates and acceptance. Learning modules on vaccine hesitancy have been developed and incorporated into didactic classes and vaccine clinics and are currently being evaluated for their effectiveness. Another limitation is that the implementation of our program by other schools will require a contract with Medicare to bill for Medicare covered services. The associated application paperwork and administrative constraints can be lengthy, cumbersome, and require considerable time for approval.

## 5. Conclusions

Vaccination billing through a web-based transaction platform provides schools of pharmacy with a net income to boost the availability and variety of vaccinations administered by student pharmacists. This incentivized and replicable vaccine billing model can be implemented by any school of pharmacy, creating an economically sustainable, community-based vaccination program that provides students with experiential training and addresses suboptimal vaccination rates of older adults and other Medicare beneficiaries.

## Figures and Tables

**Table 1:** Number and Types of Vaccines Administered and Billed at Clinic Sites from 2014–2016.

Vaccination Type	Number of Vaccinations Administered and Billed by Year (Billing System)			
	2014 (Partnering Pharmacy)	2015 (TransactRx)	2016 (TransactRx)	Total
**Influenza**				
administered	436	397	387	1220
billed	436	214	182	832
**Pneumococcal**				
administered	138	191	122	451
billed	138	191	120	449
**Tdap**				
administered	55	35	16	106
billed	55	6	3	64
**Total**				
administered	629	623	525	1777
billed	629	411	305	1345
